# Palliative care for the elderly - developing a curriculum for nursing and medical students

**DOI:** 10.1186/1471-2318-10-66

**Published:** 2010-09-20

**Authors:** Johannes M Just, Christian Schulz, Maren Bongartz, Martin W Schnell

**Affiliations:** 1 Medical Faculty Witten/Herdecke University, Institute for Ethics and Communication in Health Care, Witten, Germany; 2 Medical Faculty Dusseldorf University, Interdisciplinary Centre for Palliative Care, Dusseldorf, Germany

## Abstract

**Background:**

Delivering palliative care to elderly, dying patients is a present and future challenge. In Germany, this has been underlined by a 2009 legislation implementing palliative care as compulsory in the medical curriculum. While the number of elderly patients is increasing in many western countries multimorbidity, dementia and frailty complicate care. Teaching palliative care of the elderly to an interprofessional group of medical and nursing students can help to provide better care as acknowledged by the ministry of health and its expert panels.

In this study we researched and created an interdisciplinary curriculum focussing on the palliative care needs of the elderly which will be presented in this paper.

**Methods:**

In order to identify relevant learning goals and objectives for the curriculum, we proceeded in four subsequent stages.

We searched international literature for existing undergraduate palliative care curricula focussing on the palliative care situation of elderly patients; we searched international literature for palliative care needs of the elderly. The searches were sensitive and limited in nature. Mesh terms were used where applicable. We then presented the results to a group of geriatrics and palliative care experts for critical appraisal. Finally, the findings were transformed into a curriculum, focussing on learning goals, using the literature found.

**Results:**

The literature searches and expert feedback produced a primary body of results. The following deduction domains emerged: Geriatrics, Palliative Care, Communication & Patient Autonomy and Organisation & Social Networks. Based on these domains we developed our curriculum.

**Conclusions:**

The curriculum was successfully implemented following the Kern approach for medical curricula. The process is documented in this paper. The information given may support curriculum developers in their search for learning goals and objectives.

## Background

This article illustrates the developmental process of a curriculum on palliative care needs of the elderly. It highlights the search for relevant learning goals and objectives as this information may serve to support other curriculum developers in the process of designing similar curricula. While learning goals refer to general curriculum topics, learning objectives refer to a specific and measurable outcome [1].

Palliative Care for the elderly is a present and future challenge to health care systems around the globe as the proportion of people aged 60 years and older is growing faster than any other age group in many countries [2-4]. Traditionally, elderly people have been defined as those aged 65 and older, but the origin of this definition is unknown. An analyses performed in Japan using empirical, clinical and pathological data suggests that the term should rather apply to those 75 years and older [5].

Delivering high quality palliative care to elderly patients is complicated by different factors. Multimorbidity in general as well as dementia, frailty and other forms of functional impairment complicate palliative care for the elderly person [6-8]. Clinical difficulties are additionally escalated by demographic change as mentioned earlier. Nurses and doctors are the two principal professions who provide health care for the elderly. We know, that conflicts between the two professions can be a hindrance to adequate delivery of care [9]. At the same time, we know, that an interprofessional team approach is considered to positively influence the care situation [10-12].

Interprofessional education (IPE) can serve to improve interprofessional team skills and is best delivered at an early stage to learners in the health care sector to increase professional role understanding and to promote future teamwork [13,14]. Also, teaching Palliative Care and Geriatrics skills to health care students can be a way to improve the care situation of the elderly [3,4].

Unfortunately, the special situation of the elderly at the end of life has just recently begun to develop as a field of interest in medical education, which is partly represented by the scarcity of yet existing curricula documented later in this paper.

Against this background, we developed an interprofessional curriculum on palliative care for the elderly addressing third year nursing and medical students. Third year students were chosen to guarantee for basic knowledge in geriatrics and palliative care as well as for baseline clinical experience. The curriculum was constructed to allow for interprofessional learning experience. Following the Centre for the Advancement of Interprofessional Education, IPE occurs when two or more professions learn with, from and about each other to improve collaboration and the quality of care [15].

In order to structure the process of curriculum development, we referred to a standard approach to curriculum development [1] by Kern. Table 1 shows the six steps of curriculum development set out by Kern in relation to the sections of this paper in which they occur.

**Table 1:** Applicatio of Kern's "Six Step Approach" to this paper

Section of the paper	Step of curriculum development
Background	1. General needs assessment
	2. Characterization of learners

Results	3. Goals & Objectives (Stages 1-4)
	4. Educational strategies

Discussion	5. Implementation
	6. Evaluation & feedback.

## Methods

In the process of identifying and creating relevant learning goals and objectives for a curriculum, other curricula, general literature research and expert opinion form major sources of information. We therefore proceeded in four subsequent stages:

**Stage one: **We searched for yet existing curricula to adopt relevant goals and objectives used in these curricula.

**Stage two: **We searched for general information on the end of life needs of the elderly in order to complement the goals yet identified.

**Stage three: **Results from stage one and two were reviewed by an expert round who added further relevant information from their experience.

**Stage four: **Results from stages one, two and three are conveyed into a curriculum.

Our searches were limited literature searches in nature as grey literature, hand search and expert linking was not performed.

**Stage one, "undergraduate curricula": **we searched international literature for existing undergraduate palliative care curricula focussing on the end of life situation/palliative care in the elderly patient.

Protocol: We followed a standard protocol which is described in detail elsewhere and can be obtained from the authors [16].

Eligibility criteria: Our eligibility criteria were very wide in nature as we expected to find a very limited number of papers

• Study type: All types of studies

• Content: Describes undergraduate curricula on palliative care of the elderly.

• Language: English, German.

• Years: No restrictions

Information sources: Medline, Medline In-Process & Other Non-Indexed Citations, Embase, EBM Reviews: Cochrane DSR, ACP Journal Club, Dare, CCTR, CMR, HTA and NHSEED via OVID; AMED, ETHMED, Heclinet, gms, Springer Publisher Database and Thieme Publisher Database via Dimdi

Search: The search strategy involved 3 dimensions, "Education", "Palliative Care" and "elderly person". Mesh terms were used when possible, no limits were used. Sample search strategy for OVID:

Education *(Curriculum [Mesh] OR Short-Term Course OR education, medical, undergraduate [Mesh] OR education, nursing [Mesh] OR undergraduate teaching OR medical training) *AND Palliative Care *(Palliative Care [Mesh] OR End of life Care OR terminally ill) *AND Elderly person *(geriatrics [Mesh] OR elderly OR old)*.

Study Selection: All publications found did undergo abstract screening. Those describing undergraduate palliative care curricula for the elderly were entered in the data collection process.

Data collection Process: Data extraction was performed by two independent researchers individually and compared afterwards. A standardized sheet for data recording was used.

Data items: The following data items were extracted from the publications and illustrated in a qualitative matrix:

• "Which goals are included in the curricula?"

• "How have goals been researched?"

• "Which learning objectives are provided?"

• "Which educational strategies are used?"

**Stage two**, **"end of life needs of the elderly": **We searched international literature for general information on palliative care needs of the elderly.

Protocol: We followed a standard protocol which is described in detail elsewhere and can be obtained by the authors [16].

Eligibility criteria: In a primary search of the literature, we identified a systematic literature review [17] on the palliative care needs of elderly person published in 2003:

• Study type: all types of studies

• Content: Relates to palliative care of the elderly

• Language: English, German.

• Years: 2003 - 2009

Information sources: Medline, Medline In-Process & Other Non-Indexed Citations, Embase, EBM Reviews: Cochrane DSR, ACP Journal Club, Dare, CCTR, CMR, HTA and NHSEED via OVID; AMED, ETHMED, Heclinet, gms, Springer Publisher Database and Thieme Publisher Database via Dimdi

Search: The search strategy involved 3 dimensions, "Palliative Care", "elderly person" and "needs". Mesh terms were used when possible, no limits were used. Sample search strategy:

Palliative Care *(Palliative Care [Mesh] OR End of life Care OR terminally ill) *AND Elderly person *(geriatrics [Mesh] OR elderly OR old) *AND Needs *(needs)*

Study Selection: All publications found did undergo abstract screening. Those relating to palliative care of the elderly were entered in the data collection process.

Data collection Process: Data extraction was performed by two independent researchers individually and compared afterwards. A standardized sheet for data recording was used.

Data items: The following data items were extracted from the publications:

• "Which study design was used?"

• "What are the special needs of a dying elderly person?"

**Stage three, "expert round": **The qualitative matrices were individually presented to four experts on geriatrics and palliative care for critical appraisal. Experts were asked to comment on the results and to add additional or underrepresented issues. The expert team consisted of one expert on dementia (MScN), two experts on geriatrics (MScN, PhD) and one expert on palliative care (PhD). Experts' opinion and remarks were recorded.

**Figure 1:**
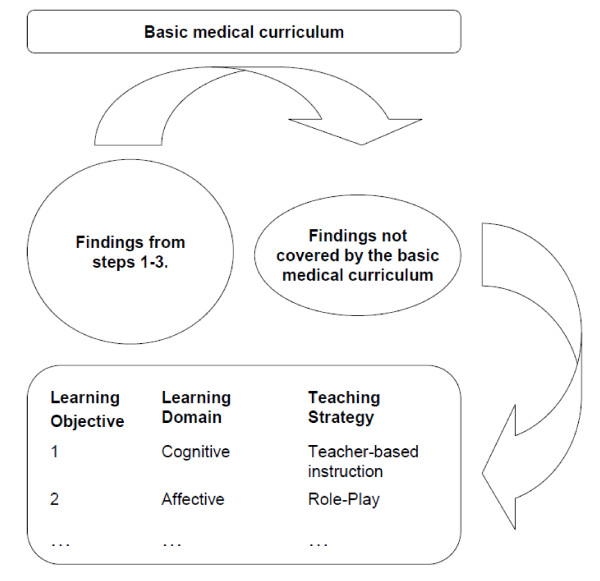
**The process from findings to learning objectives**.

**Sage four, "definition of learning objectives": **Results from stages one, two and three were fitted into a curriculum. This process was learning goal oriented and selective. The findings from steps one to three were compared with the content of the basic medical curriculum according to the medical licensure act to derive at unmet learning objectives. The remaining topics were clustered in four categories. Measurable objectives were specified. According to the learning domain (cognitive, affective, psychomotor) most dominant in each objective, learning strategies were allocated. This process is illustrated in figure 1, while an example is provided in the text to follow.

Example: Learning about how to break bad news to a patient was found to be of importance. Additionally, in the situation of the elderly, often the diagnosis is less relevant to the patient than the course of suffering to be expected. While "Breaking Bad News" is already part of the basic medical curriculum, the second factor is not. The corresponding learning objective can be formulated as:

After the seminar, the student knows about the specific pitfalls when breaking bad news to an elderly person. This learning objective mainly involves cognitive aspects of teaching. Therefore teacher-based instruction and case-based learning are used as a means of teaching methodology.

## Results

The following section will summarize the curriculum development steps three and

four after Kern. Step three comprises the development, definition and categorization of learning objectives, while step four identifies the specific educational strategies for those learning objectives [1].

**Figure 2:**
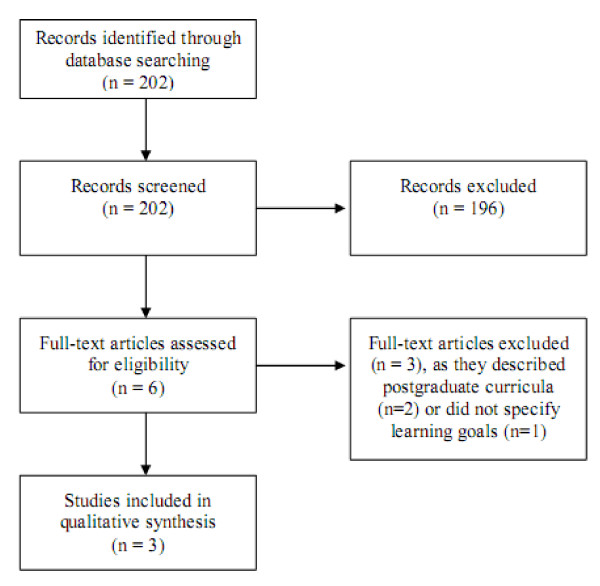
**Flow-diagram literature search curricula**.

**Results stage one, "undergraduate curricula": **The search was first performed in February 2008 and last updated in July 2009 and produced 202 results. The process of study selection is shown in figure 2.

Identified learning goals were clustered into four main categories: (1) Geriatrics, (2) Palliative Care, (3) Communication & Patient Autonomy and (4) Organisation & Social networks. A detailed list of learning goals can be found in table 2. Two articles included indicated that their respective learning goals were based on expert advice only [18,19]. One article did not comment on a search strategy for learning goals at all [20]. No learning objectives were presented in any of the articles.

**Table 2:** Identified learning goals

Field	Learning goals
**Geriatrics**	○ Geriatric assessment [18]. ○ Importance of interdisciplinary team approach [18].

**Palliative Care**	○ Symptom management [18-20]. ○ Systematic pain management [18,19]. ○ Palliative Assessment [18]. ○ Professional self care [18].

**Communication and patient autonomy**	○ Communication with patients and relatives [20]. ○ Grief [20]. ○ Ethical aspects [20]. ○ Breaking Bad News [18,19]. ○ Advance directives [19].

**Organisation and** **social networks**	○ Administration of care [20]

The educational strategies used included: Power point presentation, case studies, reference articles, role-play and reflection as well as discussion rounds.

**Figure 3:**
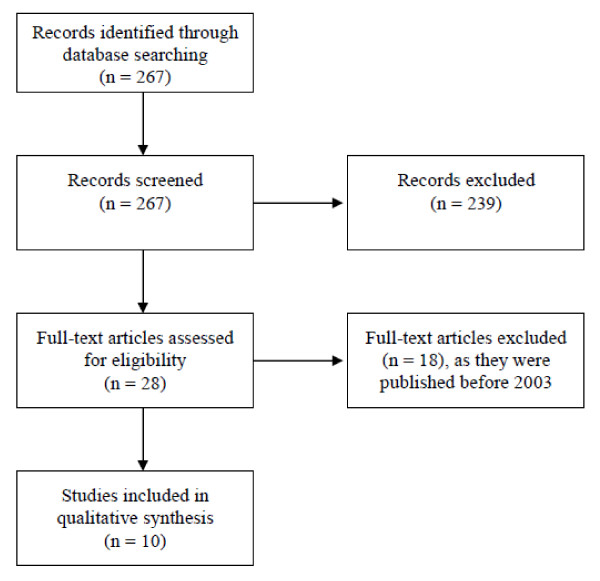
**Flow-diagram literature search needs**.

**Results stage two**, **"end of life needs of the elderly": **The search was first performed in March 2008 and last updated in August 2009 and produced 267 results. The process of study selection is shown in figure 3.

The results included five expert articles [6-8,21,22], two qualitative interview studies [23,24], two literature reviews [11,17] and one prospective questionnaire study [25]. Topics were clustered under the existing categories from stage one. A detailed list of topics can be found in table 3.

**Table 3:** Results of literature search "end of life needs of the elderly"

Field	Topics
**Geriatrics**	○ Holistic approach is important [11]. ○ Even though a potential life limiting disease is present, common symptoms of old age like bladder control difficulties or impaired vision can cumulatively have a considerable negative effect on quality of life [25]. ○ Geriatric assessment and knowledge of common problems like falls, delir, fragility, incontinence and especially dementia [6,21]. ○ Multimorbidity as a challenge for prognosis, e.g. leading to inefficient timing of hospice admission [7,21].

**Palliative Care**	○ Personal, psychological and existential issues have to be addressed [11,17,23]. ○ Cultural influence on personal, psychological and existential issues has to be considered [11]. ○ Efficient pain control is crucial [17,24]. ○ Liberty from pain is necessary for personal, existential and spiritual issues to be addressed [24]. ○ Patients aged 70 and above need support in dealing with existential and spiritual issues despite their age [25]. ○ While treating symptoms, the fragile equilibrium of an elderly person's physiology has to be kept in mind at all times [7,8,21].

**Communication and** **patient autonomy**	○ Effective communication between caretakers and patient leads towards improved health outcomes and quality of care [6,8,17]. ○ In dementia, it is crucial to plan and implement a plan of care while the patient is still able to do so. The family should be involved at an early stage [6]. ○ Communication with the patient's family is a necessary part of care work [22]. ○ The interprofessional team approach is important [11,22].

**Organisation and** **social networks**	○ Unlike patients in their mid-life segment, elderly people often have a weak social network. This leads to a lack of close persons who can be addressed with personal, existential and spiritual issues, also less support in organisational issues e.g. through family members is accessible [8]. ○ A key worker can help improve the organisation of care [17]. ○ Patients have to be educated and aided by caretakers to develop and implement a thorough plan of care. Advance directives should be made [6-8]. ○ Elderly patients do wish to have more contact with their caretakers but often do not ask for it for fear of being a burden [23,25].

**Results stage three, "expert round": **The qualitative matrices were presented to all four experts in June 2008 during four individual two-hour sessions.

As a result, all experts approved of the stage one and two results and gave the following additional input as displayed in table 4.

**Table 4:** Expert round

Field	Comments
**Geriatrics**	○ In patients with dementia, perform pain assessment with visual instruments. ○ Relevance of key worker for patients with dementia is high. ○ In dementia, challenging behaviour can be interpreted as an attempt to communication. ○ The use of hospices for patients suffering from dementia is questionable.

**Palliative Care**	○ Unnecessary interventions and waiting periods may consume precious time at the end of life.

**Communication and** **patient autonomy**	○ When breaking bad news to an elderly person, the diagnosis is in many cases less relevant to the patient than the course of suffering to be expected. ○ General pitfalls, like impaired hearing or understanding of complex contexts have to be kept in mind.

**Organisation and** **social networks**	○. Negative aspects on institutionalization may occur (loss of individuality and freedom).

Results stage four, "definition of learning objectives":

The final list of learning goals from all four main categories was transformed into individual learning objectives. Educational strategies related to the respective learning domain (cognitive, affective and psychomotor) were defined for each learning objective. Results are displayed in tables 5, 6, 7 and 8.

**Table 5:** Curriculum part A - Geriatrics

**Learning Goal **(recommended reading)	Learning Objective	Educational Strategy	Learning Domain
**Multimorbidity** [6,7,21]	-Student knows about the challenge of prognosis in the elderly	Teacher-based instruction	Cognitive
	-Student knows about negative effects of hospitalisation	Case-based learning	

**Geriatric Assessment** [6,21]	-Student knows about the challenge of symptom control in the elderly	Teacher-based instruction	Cognitive
	-student is familiar with standard tools of geriatric assessment, especially visual pain assessment tools	Case-based learning	

**Burden of old age** [25]	-Student knows and accepts the impairment in life quality caused by seemingly "trifle" diseases	Teacher-based instruction Role Play	Cognitive Affective

**Table 6:** Curriculum part B - Palliative Care

Learning Goal (recommended reading)	Learning Objective	Educational Strategy	Learning Domain
**Holistic Care** [11,17,23,25]	-Student knows about and accepts the importance of personal, psychological, existential and spiritual issues at the end of life	Teacher-based instruction Discussion Case-based learning	Cognitive Affective
	-Student knows about and accepts the fact, that the issues stated above vary widely due to cultural influence - Student knows about and accepts the fact that elderly people have a need to discuss the		
	issues stated above, despite their old age		

**Pain control** [17,24]	-student knows and accepts the point that efficient pain control is crucial to successful palliative care	Teacher-based instruction	Cognitive
	- student knows and accepts the point that freedom of pain is important for personal, psychological, existential and spiritual issues at the end of life to be addressed	Discussion	Affective

**Burden of old age 2 **[7,8,21]	-Student knows that while treating symptoms in the elderly, the fragile equilibrium of an elderly persons physiology has to be considered and protected at all cost	Teacher-based instruction Case-based learning	Cognitive

**Table 7:** Curriculum part C- Communication and Patient Autonomy

Learning Goal (recommended reading)	Learning Objective	Educational Strategy	Learning Domain
**Relevance of communication** [6,8,17]	-Student knows that effective communication between caretakers and patient as well as relatives leads towards improved health outcomes and quality of care	Teacher-based instruction Discussion	Cognitive Affective

**Interprofessional team approach** [11,22]	-Student knows about and accepts relevance of the interdisciplinary team approach	Teacher-based instruction Discussion	Cognitive Affective

**Advance directives** [6]	-Student knows about and accepts the importance of advance directives, especially in patients with dementia.	Teacher-based instruction Discussion	Cognitive Affective

**Breaking Bad News** (Expert advice)	-Student knows about the specific pitfalls when breaking bad news to an elderly person.	Teacher-based instruction Case-based learning	Cognitive

**Table 8:** Curriculum part 4 D - Organisation and Social Networks

Learning Goal (recommended reading)	Learning Objective	Educational Strategy	Learning Domain
**Social networks** [8]	-Student knows about and accepts the effect on an elderly person's life caused by a loss of social networks.	Teacher-based instruction Discussion	Cognitive Affective

**Plan of care** [6-8,17]	-Student understands and accepts the relevance of developing a plan of care and making advance directives	Teacher-based instruction	Cognitive
	-Student knows that a key worker can help achieve this	Discussion	Affective

**Need for care** [23,25]	-Student knows and accepts that elderly patients do wish to have more contact with their caretakers but often do not ask for it for fear of being a burden	Teacher-based instruction Discussion	Cognitive Affective

The Curriculum was taught on two consecutive days with a total delivery time of 12 hours to ten medical students and ten nursing students. A teacher-student ratio between 1:5 and 1:10 is suggested for successful IPE as discussed by Oandasan and Reeves in 2005 [26]. Three experts, specialized in the field of palliative care, geriatrics and communication science, respectively, conducted the curriculum.

## Discussion

The goal of this study was to establish an interprofessional curriculum on palliative care for the elderly. We utilized the "Six-Step-Approach" by Kern and derived at a four domains curriculum, including 18 learning objectives with a total of 12 teaching units. While other approaches towards curriculum development exist, we used Kerns approach as it is a standard in the field and can be applied easily. The curriculum is comparably short - this only makes sense in the context of the basic medical curriculum expanding it for the aspect of taking care of the elderly at the end of life.

The curriculum was taught to an interprofessional group of third year nursing and medical students (n = 20 [10/10]). IPE is best delivered early to learners in the health care sector [13,14]. Still, we choose third year students. This was done as our curriculum is complementary in nature, therefore participants had to have passed through the curricula on palliative care and the courses on geriatrics beforehand. This might have lessened a possible positive effect of IPE.

The increasing need for IPE and education on palliative care is acknowledged on a national level for instance by the German government [27,28] as well as on an international level by the WHO [4,29]. Teaching palliative care for the elderly to students is important and can improve the quality of care [3,4], especially if it is done in an interprofessional way [10,11], like in the case of our curriculum. Combining the fields of palliative care and geriatrics is particularly generative when using IPE as both take pride in and value the interprofessional team approach [10], thereby forming a synergistic effect. The combination therefore makes sense; still, our search displayed a scarcity of well documented curricula on palliative care of the elderly.

Bickel-Swenson in her 2007 systematic review finds, that there is a considerable lack of standardisation in medical education related to end-of-life care [30]. Lloyd-Williams has found the same situation in his 2004 review of the European situation [31]. Official consensus guidelines and recommendations on Undergraduate Palliative Care Education exist both on the European [32] and German National level [33,34], but their primary focus is on defining required core competencies for palliative care education. What is missing is an answer to the how of teaching and training of those competencies as well as clearly defined learning objectives. By this study, we hope to give a best practice example and to start a more public exchange process between curriculum developers. To our knowledge, this is the first interprofessional undergraduate curriculum on palliative care of the elderly including learning objectives as well as teaching methods, reported in the literature.

However, several limitations to our methodology apply. The search strategy for the needs of elderly patients could be improved as the search term "needs" combined with the AND operator possibly limited our results. This was done in the face of limited time and resources while trying not to miss out on relevant publications. Also, some curricula may not have been reported in the literature searched. Additionally, we have to take into account a publication bias as the literature searches conducted have been limited due to lack of time and resources - grey literature search, hand search and expert linking were not performed.

Still, results can be generalized when keeping the limitations mentioned in mind. As the field of palliative care for the elderly is an emerging field in the context of palliative care in general, curricula will have to be updated regularly to be in touch with the most recent body of evidence.

Eventually, Kerns' steps 5 and 6 have to be acknowledged. Step5, Implementation, involves organisation and identification of resources (such as personnel, time, facilities and costs) as well as possible pitfalls, especially when interprofessional projects and therefore different departments are involved. Step 6, evaluation, is vital for further curricular development as it helps to improve quality of teaching as well as choice of learning goals and objectives. We evaluated our curriculum using a simulated practice setting and a randomized controlled trial methodology. The evaluation showed an improvement in quality of jointly formulated learning objectives as well as a moderate effect towards change in interprofessional communication style. This process is documented in detail elsewhere (Just JM, Schulz C et al.: Exploring Effects of Interprofessional Education on Undergraduate Students' Behaviour - A Randomized Controlled Trial. Journal of Research in Interprofessional Education, accepted for publication).

## Conclusions

Taking care of dying, elderly patients is a challenge in itself, a challenge that will grow in extent due to population development as shown in the background section. Thus healthcare professionals need to be up for the task. Taking part in a curriculum like the one described in this paper might help them to do so.

We therefore encourage curriculum developers to work on an individual curriculum on palliative care of the elderly, adjusted to their institution, using support by Kern's "Six-Step-Approach" and the information given in this paper.

## Abbreviations

IC: Interprofessional Curriculum; IPE: Interprofesional Education.

## Competing interests

The authors declare that they have no competing interests

## Authors' contributions

JMJ was responsible for conception and content of the paper, MB made substantial contributions to acquisition of data, CS has been involved in drafting the manuscript and revising it critically, MWS revised it critically and gave final approval of the version to be published. All authors read and approve the final draft.

## Pre-publication history

The pre-publication history for this paper can be accessed here:

http://www.biomedcentral.com/1471-2318/10/66/prepub
